# Supplementary material for: Sarcomeric remodelling in human heart failure unraveled by single molecule long read sequencing
Source: EMBO Mol Med. 2026 Jan 13;18(2):824–45. doi: 10.1038/s44321-025-00370-9 (PMC12905364; doi:10.1038/s44321-025-00370-9)
Supplement: Supplementary file 1 — Appendix [file 44321_2025_370_MOESM1_ESM.pdf]

Appendix

Table of content:

- **Appendix Figure S1: Exon structure of transcript isoforms of the tropomyosin family (p.1)**
- **Appendix Figure S2: Protein alignment per exon of transcript isoforms of the tropomyosin family (p.1)**
- **Appendix Table S1: Basic proband phenotypes (p. 5)**

Appendix Figure S1: Exon structure of transcript isoforms of the tropomyosin family

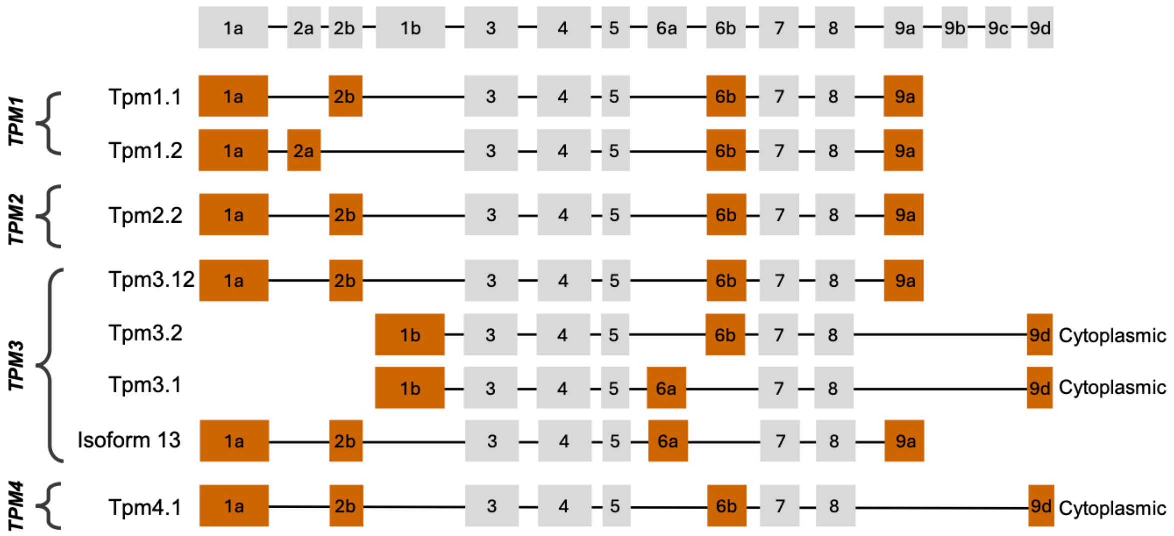

Appendix Figure S1: Exon structure of transcript isoforms of the tropomyosin family. Exon structure for the four major tropomyosin genes (*TPM1*, *TPM2*, *TPM3*, and *TPM4*) and their isoforms are shown. These isoforms arise through alternative splicing, essentially four key exons—1, 2, 6, and 9 (marked in orange).

Appendix Figure S2: Protein alignment per exon of transcript isoforms of the tropomyosin family

amino acids in different chemical groups: ■

amino acids in the same chemical group: ■

Exon 1a

Tpm1.1	(TPM1-207)	MDAIKKKMQMLKLDKENALDRAEQAEADKKAAEDRSKQ
Tpm1.2	(TPM1-229)	MDAIKKKMQMLKLDKENALDRAEQAEADKKAAEDRSKQ
Tpm2.2	(TPM2-209)	MDAIKKKMQMLKLDKENAIDRAEQAEADKKQAEDRCKQ
Tpm3.12	(TPM3-224)	MMEAIKKKMQMLKLDKENALDRAEQAEAEQKQAEERSKQ

Tpm3.2	(TPM3-206)		--------------------------------------
Tpm3.1	(TPM3-212)		--------------------------------------
Isoform 13	(TPM3-210)		MEAIKKKMQMLKLDKENALDRAEQAEAEQKQAEERSKQ
Tpm4.1	(TPM4-221)		MEAIKKKMQMLKLDKENAIDRAEQAEADKKAAEDKCKQ

Exon 1b

Tpm1.1	(TPM1-207)		--
Tpm1.2	(TPM1-229)		--
Tpm2.2	(TPM2-209)		--
Tpm3.12	(TPM3-224)		--
Tpm3.2	(TPM3-206)		MAGITTIEAVKRKIQVLQQQADDAEERAERLQREVEGERRAREQ
Tpm3.1	(TPM3-212)		MAGITTIEAVKRKIQVLQQQADDAEERAERLQREVEGERRAREQ
Isoform 13	(TPM3-210)		--
Tpm4.1	(TPM4-221)		--

Exon 2a/b

Tpm1.1	(TPM1-207)		LEDELVSLQKKLKGTEDELDKYSEALKDAQEKLELAEKKATD
Tpm1.2	(TPM1-229)	(2a)	LEEDIAAKEKLLRVSEDERDRVLEELHKAEDSLLAAEEAAAK
Tpm2.2	(TPM2-209)		LEEEQQALQKKLKGTEDEVEKYSESVKEAQEKLEQAEKKATD
Tpm3.12	(TPM3-224)		LEDELAAMQKKLKGTEDELDKYSEALKDAQEKLELAEKKAAD
Tpm3.2	(TPM3-206)		--
Tpm3.1	(TPM3-212)		--
Isoform 13	(TPM3-210)		LEDELAAMQKKLKGTEDELDKYSEALKDAQEKLELAEKKAAD
Tpm4.1	(TPM4-221)		VEEELTHLQKKLKGTEDELDKYSEDLKDAQEKLELTEKKASD

Exon 3

Tpm1.1	(TPM1-207)		AEADVASLNRRIQLVEEELDRAQERLATALQKLEEAEKAADESER
Tpm1.2	(TPM1-229)		AEADVASLNRRIQLVEEELDRAQERLATALQKLEEAEKAADESER
Tpm2.2	(TPM2-209)		AEADVASLNRRIQLVEEELDRAQERLATALQKLEEAEKAADESER
Tpm3.12	(TPM3-224)		AEAEVASLNRRIQLVEEELDRAQERLATALQKLEEAEKAADESER
Tpm3.2	(TPM3-206)		AEAEVASLNRRIQLVEEELDRAQERLATALQKLEEAEKAADESER
Tpm3.1	(TPM3-212)		AEAEVASLNRRIQLVEEELDRAQERLATALQKLEEAEKAADESER
Isoform 13	(TPM3-210)		AEAEVASLNRRIQLVEEELDRAQERLATALQKLEEAEKAADESER
Tpm4.1	(TPM4-221)		AEGDVAALNRRIQLVEEELDRAQERLATALQKLEEAEKAADESER

Exon 4

Tpm1.1	(TPM1-207)		GMKVIESRAQKDEEKMEIQEIQLKEAKHIAEDADRKYEE
Tpm1.2	(TPM1-229)		GMKVIESRAQKDEEKMEIQEIQLKEAKHIAEDADRKYEE
Tpm2.2	(TPM2-209)		GMKVIENRAMKDEEKMELQEMQLKEAKHIAEDSDRKYEE
Tpm3.12	(TPM3-224)		GMKVIENRALKDEEKMELQEIQLKEAKHIAEEADRKYEE
Tpm3.2	(TPM3-206)		GMKVIENRALKDEEKMELQEIQLKEAKHIAEEADRKYEE
Tpm3.1	(TPM3-212)		GMKVIENRALKDEEKMELQEIQLKEAKHIAEEADRKYEE
Isoform 13	(TPM3-210)		GMKVIENRALKDEEKMELQEIQLKEAKHIAEEADRKYEE
Tpm4.1	(TPM4-221)		GMKVIENRAMKDEEKMEIQEMQLKEAKHIAEEADRKYEE

Exon 5

Tpm1.1	(TPM1-207)		VARKLVIIESDLERAEERAELSEG
Tpm1.2	(TPM1-229)		VARKLVIIESDLERAEERAELSEG
Tpm2.2	(TPM2-209)		VARKLVILEGELERSEERAEVAES
Tpm3.12	(TPM3-224)		VARKLVIIEGDLERTEERAELAES
Tpm3.2	(TPM3-206)		VARKLVIIEGDLERTEERAELAES
Tpm3.1	(TPM3-212)		VARKLVIIEGDLERTEERAELAES
Isoform 13	(TPM3-210)		VARKLVIIEGDLERTEERAELAES
Tpm4.1	(TPM4-221)		VARKLVILEGELERAEERAEVSEL

Exon 6b/a

Tpm1.1	(TPM1-207)		KCAELEEELKTVTNNLKSLEAQAEK
Tpm1.2	(TPM1-229)		KCAELEEELKTVTNNLKSLEAQAEK
Tpm2.2	(TPM2-209)		KCGDLEEELKIVTNNLKSLEAQADK
Tpm3.12	(TPM3-224)		KCSELEEELKNVTNNLKSLEAQAEK
Tpm3.2	(TPM3-206)		KCSELEEELKNVTNNLKSLEAQAEK
Tpm3.1	(TPM3-212)	(6a)	RCREMDEQIRLMDQNLKCLSAAEEK
Isoform 13	(TPM3-210)	(6a)	RCREMDEQIRLMDQNLKCLSAAEEK
Tpm4.1	(TPM4-221)		KCGDLEEELKNVTNNLKSLEAASEK

Exon 7

Tpm1.1	(TPM1-207)		YSQKEDRYEEEIKVLSDKLKE
Tpm1.2	(TPM1-229)		YSQKEDRYEEEIKVLSDKLKE
Tpm2.2	(TPM2-209)		YSTKEDKYEEEIKLLEEKLKE
Tpm3.12	(TPM3-224)		YSQKEDKYEEEIKILTDKLKE

Tpm3.2	(TPM3-206)	YSQKEDKYEEEIKILTDKLKE
Tpm3.1	(TPM3-212)	YSQKEDKYEEEIKILTDKLKE
Isoform 13	(TPM3-210)	YSQKEDKYEEEIKILTDKLKE
Tpm4.1	(TPM4-221)	YSEKEDKYEEEIKLLSDKLKE

Exon 8

Tpm1.1	(TPM1-207)	AETRAEFAERSVTKLEKSIDDLED
Tpm1.2	(TPM1-229)	AETRAEFAERSVTKLEKSIDDLED
Tpm2.2	(TPM2-209)	AETRAEFAERSVAKLEKTIDDLED
Tpm3.12	(TPM3-224)	AETRAEFAERSVAKLEKTIDDLED
Tpm3.2	(TPM3-206)	AETRAEFAERSVAKLEKTIDDLED
Tpm3.1	(TPM3-212)	AETRAEFAERSVAKLEKTIDDLED
Isoform 13	(TPM3-210)	AETRAEFAERSVAKLEKTIDDLED
Tpm4.1	(TPM4-221)	AETRAEFAERTVAKLEKTIDDLEE

Exon 9a

Tpm1.1	(TPM1-207)	ELYAQKLKYKAISEELDHALNDMTSI
Tpm1.2	(TPM1-229)	ELYAQKLKYKAISEELDHALNDMTSI
Tpm2.2	(TPM2-209)	EVYAQKMKYKAISEELDNALNDITSL
Tpm3.12	(TPM3-224)	ELYAQKLKYKAISEELDHALNDMTSI
Tpm3.2	(TPM3-206)	--------------------------
Tpm3.1	(TPM3-212)	--------------------------
Isoform 13	(TPM3-210)	ELYAQKLKYKAISEELDHALNDMTSI
Tpm4.1	(TPM4-221)	--------------------------

Exon 9d

Tpm1.1	(TPM1-207)	--------------------------
Tpm1.2	(TPM1-229)	--------------------------
Tpm2.2	(TPM2-209)	--------------------------
Tpm3.12	(TPM3-224)	--------------------------
Tpm3.2	(TPM3-206)	KLKCTKEEHLCTQRMLDQTLLDLNEM
Tpm3.1	(TPM3-212)	KLKCTKEEHLCTQRMLDQTLLDLNEM
Isoform 13	(TPM3-210)	--------------------------
Tpm4.1	(TPM4-221)	KLAQAKEENVGLHQTLDQTLNELNCI

Appendix Figure S2: Protein alignment per exon of transcript isoforms of the tropomyosin family. Major tropomyosin isoforms have been aligned based on their protein sequence as predicted by the long-read sequence. Amino acids marked in grey belong to the same chemical group, whereas the ones marked in turquoise are from a different chemical group

Appendix Table S1: Basic proband phenotypes

group	mean age	male sex
CTRL	48 (± 14,19)	n=9 (70%)
DCM	45 (± 14,49)	n=7 (70%)
ICM	57 (± 3,29)	n=9 (90%)
