# Supplementary material for: Sarcomeric remodelling in human heart failure unraveled by single molecule long read sequencing
Source: EMBO Mol Med. 2026 Jan 13;18(2):824–45. doi: 10.1038/s44321-025-00370-9 (PMC12905364; doi:10.1038/s44321-025-00370-9)
Supplement: Supplementary file 10 — Movie EV4 [file 44321_2025_370_MOESM10_ESM.zip › EMM-2025-21535_MovieEV4_Legend.docx]

**Legend: Expanded View Movie EV4**

The sliding velocity of rhodamine-phalloidin-labeled actin filaments or reconstituted thin filaments (labelled actin + tropomyosin + troponin) regulated by calcium, moved by beta-cardiac myosin HMM immobilised on a nitrocellulose covered glass surface was observed with TIRF microscopy (Olympus). Videos were recorded with Olympus xcellence rt software with either one frame per second or two frames per second (FPS). Analysis of videos was done with FijiREF1 and the plugın wrMTrckREF2 with the following settings: Scale known distance: 1 px= 146.5 nm ; Min= 5 –Max= 1,000 ; Max velocity= 30 ; Max area change= 50 ; Min track length= 10 ; Bend= 2 ; FPS= 2 or FPS= 1 (dependent on the video) as previously described by *Schindelin, J et al. Fiji: an open-source platform for biological-image analysis. Nat Methods 9, 676-682 (2012) ; Nussbaum-Kramer CI, Neto MF, Brielmann RM, Pederson JS, Morimoto RI. Investigating the spreading and toxicity of prion-like proteins using metazoan model organism C. elegans. J. Vis. Exp. 52321 (2015)*.

**EMM-2025-21535_MovieEV4:** Movie of the sliding velocity of TPM1.1 at pCa 4
