# Supplementary figures and images for: Sarcomeric remodelling in human heart failure unraveled by single molecule long read sequencing
Source: EMBO Mol Med. 2026 Jan 13;18(2):824–45. doi: 10.1038/s44321-025-00370-9 (PMC12905364; doi:10.1038/s44321-025-00370-9)

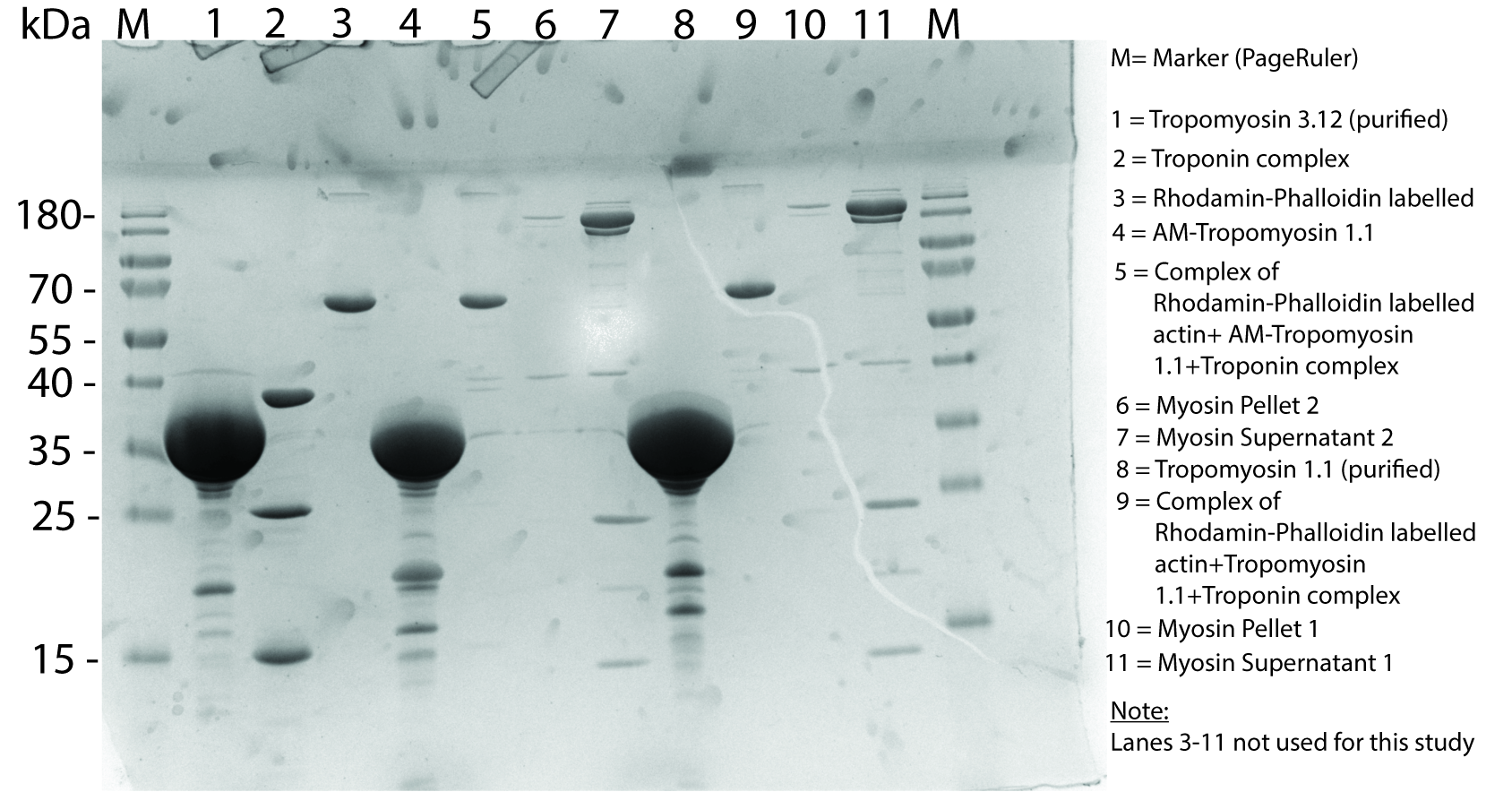

Supplement: Supplementary file 13 — Source data Fig. 7 [file 44321_2025_370_MOESM13_ESM.zip › Figure7/7C/7C.tif]
