# Supplementary material for: Sarcomeric remodelling in human heart failure unraveled by single molecule long read sequencing
Source: EMBO Mol Med. 2026 Jan 13;18(2):824–45. doi: 10.1038/s44321-025-00370-9 (PMC12905364; doi:10.1038/s44321-025-00370-9)
Supplement: Supplementary file 14 — Expanded View Figures [file 44321_2025_370_MOESM14_ESM.pdf]

Expanded View Figures

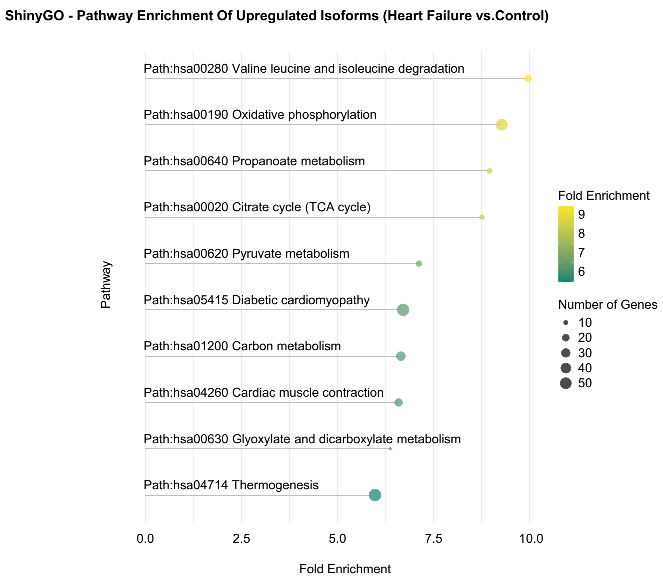

Figure EV1. Pathway enrichment of upregulated isoforms (heart failure vs. control).

We performed a pathway analysis (ShinyGO) on upregulated isoforms in heart failure (DCM + ICM) vs. controls. Color scheme visualizes the fold enrichment with darker color being stronger enriched, while the size of the bullet represents the number of shared genes in the respective pathway. Data for running the analysis is provided in Dataset EV1.

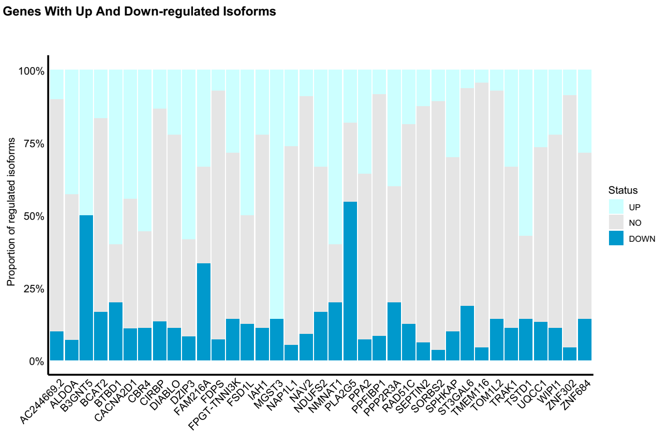

Figure EV2. Genes with up- and down-regulated Isoforms (heart failure vs. control).

Shown are the number of upregulated (“UP”), downregulated (“DOWN”), and not regulated (“NO”) per gene for coding genes which show regulated transcript isoforms in opposing directions.

© The Author(s)

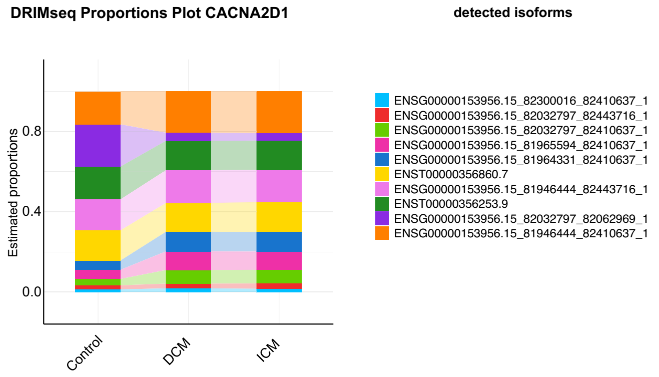

Figure EV3. DRIMseq proportions plot.

Overview of detected isoforms for *CACNA2D1* and their proportional contribution to all detected isoforms of the gene.

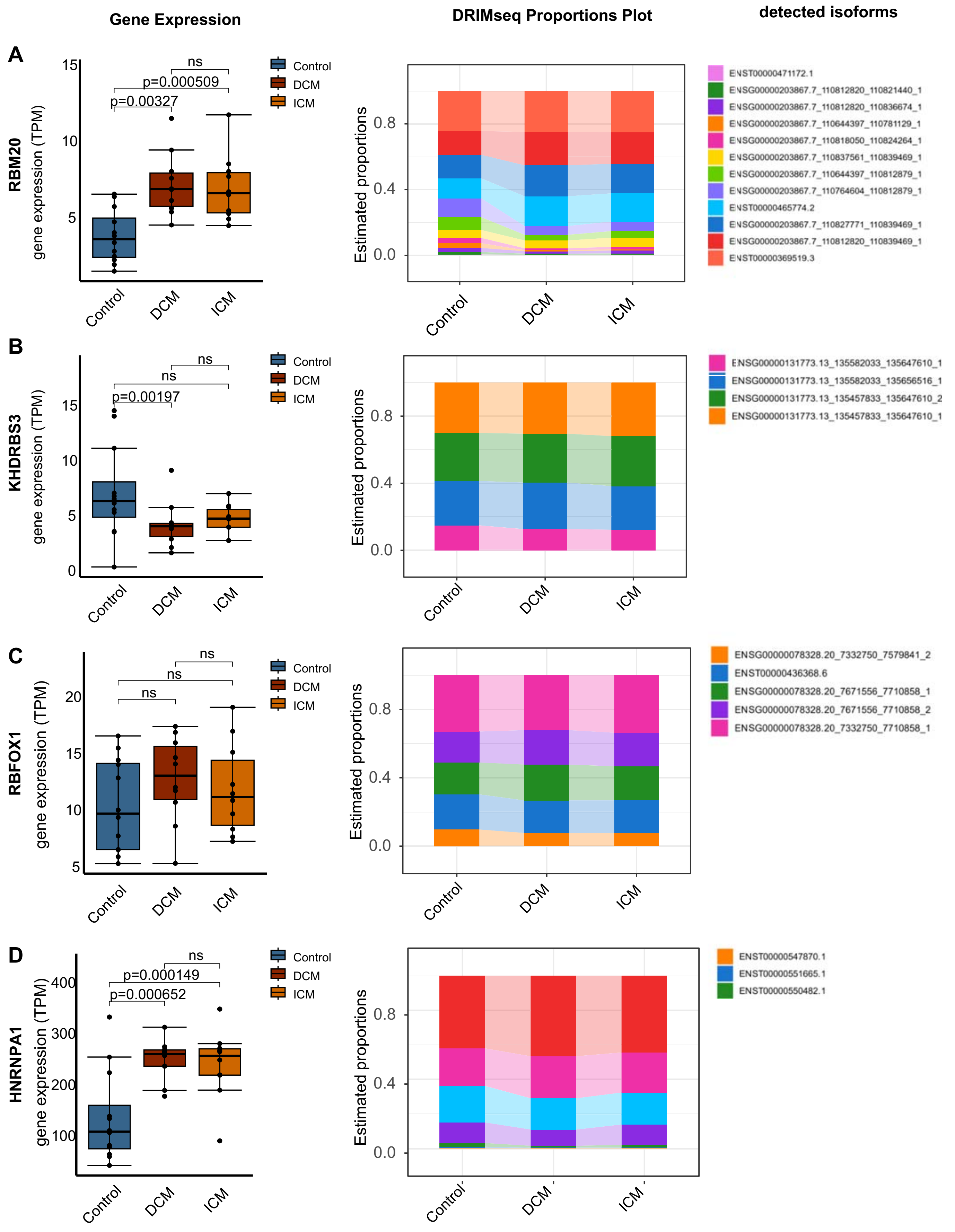
Gene Expression
DRIMseq Proportions Plot
detected isoforms
A
RBM20
gene expression (TPM)
ns
p=0.000509
p=0.00327
Control
DCM
ICM
Estimated proportions
ENST00000471172.1
ENSG00000203867.7_110812820_110821440_1
ENSG00000203867.7_110812820_110836674_1
ENSG00000203867.7_110644397_110781129_1
ENSG00000203867.7_110818050_110824264_1
ENSG00000203867.7_110837561_110839469_1
ENSG00000203867.7_110644397_110812879_1
ENSG00000203867.7_110764604_110812879_1
ENST00000465774.2
ENSG00000203867.7_110827771_110839469_1
ENSG00000203867.7_110812820_110839469_1
ENST00000369519.3
B
KHDRBS3
gene expression (TPM)
ns
ns
p=0.00197
Control
DCM
ICM
Estimated proportions
ENSG00000131773.13_135582033_135647610_1
ENSG00000131773.13_135582033_135656516_1
ENSG00000131773.13_135457833_135647610_2
ENSG00000131773.13_135457833_135647610_1
C
RBFOX1
gene expression (TPM)
ns
ns
ns
Control
DCM
ICM
Estimated proportions
ENSG00000078328.20_7332750_7579841_2
ENST00000436368.6
ENSG00000078328.20_7671556_7710858_1
ENSG00000078328.20_7671556_7710858_2
ENSG00000078328.20_7332750_7710858_1
D
HNRNPA1
gene expression (TPM)
ns
p=0.000149
p=0.000652
Control
DCM
ICM
Estimated proportions
ENST00000547870.1
ENST00000551665.1
ENST00000550482.1

© The Author(s)

Figure EV4. Gene expression and DRIMseq proportions plot for important splice factor genes.

Gene expression in transcript per million (TPM) for CTRL ($n = 13$), DCM ($n = 10$), and ICM ($n = 10$) probes. Box plots in the left panel show gene expression of the splice factors RBM20 (**A**), KHDRBS3 (**B**), RBFOX1 (**C**), and HNRNP A1 (**D**). The right panel displays an overview of the proportion of the detected transcript isoforms of the respective genes, as determined by DRIMseq. Values ranged as following for *RBM20*-CTRL (min = 0.355; max = 6.36; median = 3.19; q1 = 2.05; q3 = 4.55; p95 = 6.32); *RBM20*-DCM (min = 4.34; max = 11.3; median = 6.69; q1 = 5.57; q3 = 7.74; p95 = 11.0); *RBM20*-ICM (min = 4.30; max = 11.6; median = 6.42; q1 = 5.13; q3 = 7.76; p95 = 11.0); *KHDRBS3*-CTRL (min = 0.112; max = 14.3; median = 6.28; q1 = 5.06; q3 = 10.9; p95 = 14.2); *KHDRBS3*-DCM (min = 1.39; max = 8.88; median = 4.07; q1 = 2.88; q3 = 4.07; p95 = 8.27); *KHDRBS3*-ICM (min = 2.52; max = 6.77; median = 4.50; q1 = 3.72; q3 = 5.32; p95 = 6.57); *RBFOX1*-CTRL (min = 5.05; max = 16.3; median = 9.78; q1 = 6.28; q3 = 13.8; p95 = 16.1); *RBFOX1*-DCM (min = 5.07; max = 17.2; median = 12.8; q1 = 10.7; q3 = 15.4; p95 = 17.1); *RBFOX1*-ICM (min = 7.01; max = 18.9; median = 10.9; q1 = 8.43; q3 = 14.2; p95 = 18.5); *HNRNPA1*-CTRL (min = 36.6; max = 329.0; median = 105.0; q1 = 72.6; q3 = 218.0; p95 = 329.0); *HNRNPA1*-DCM (min = 172.0; max = 307.0; median = 255.0; q1 = 231.0; q3 = 263.0; p95 = 301.0); *HNRNPA1*-ICM (min = 84.6; max = 343.0; median = 251.0; q1 = 214.0; q3 = 265.0; p95 = 331.0).

© The Author(s)

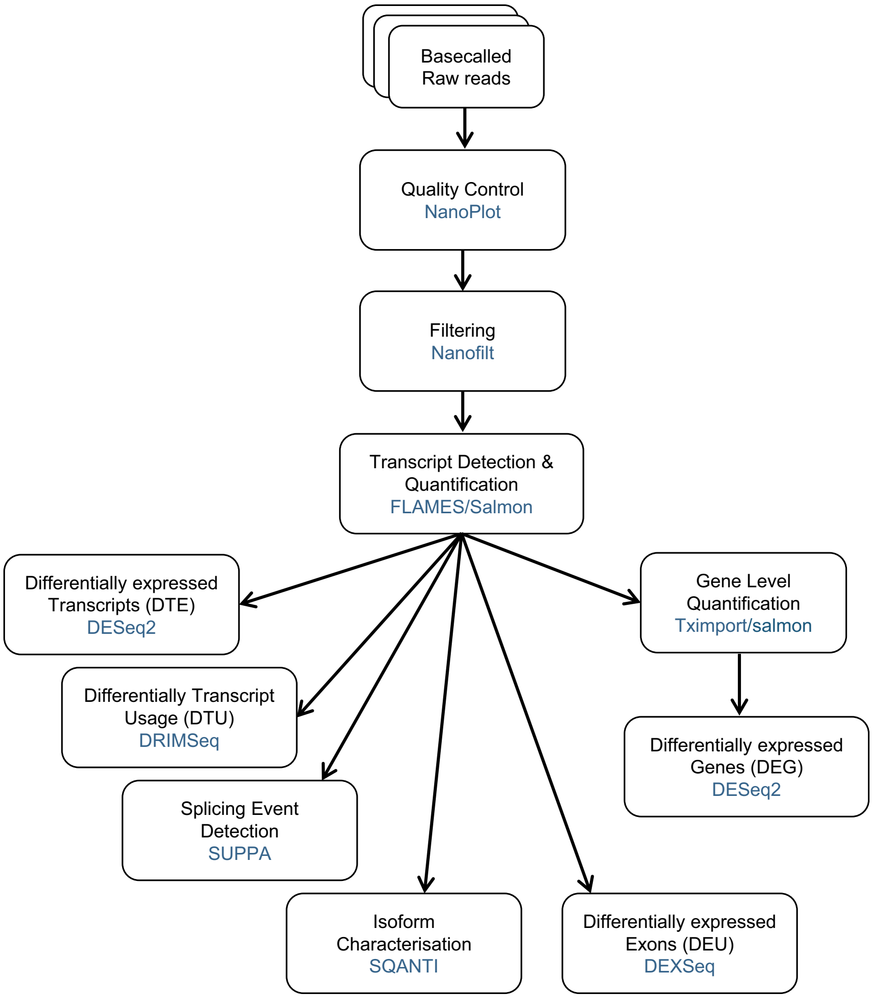

Figure EV5. Analysis pipeline.

Scheme is showing the workflow of the data analysis and list used tools per analysis.

© The Author(s)
